# Association of Motor Function With Cognitive Trajectories and Structural Brain Differences: A Community-Based Cohort Study

**DOI:** 10.1212/WNL.0000000000207745

**Published:** 2023-10-24

**Authors:** Zhangyu Wang, Jiao Wang, Jie Guo, Abigail Dove, Konstantinos Arfanakis, Xiuying Qi, David A. Bennett, Weili Xu

**Affiliations:** From the Department of Epidemiology and Biostatistics (Z.W., J.W., X.Q., W.X.), School of Public Health, Tianjin Medical University, China; Aging Research Center, Department of Neurobiology (J.G., A.D., W.X.), Care Sciences and Society, Karolinska Institutet, Stockholm, Sweden; Rush Alzheimer's Disease Center (K.A., D.A.B.), Rush University Medical Center, Chicago, IL; and Department of Biomedical Engineering (K.A.), Illinois Institute of Technology, Chicago.

## Abstract

**Background and Objectives:**

The association of motor function with cognitive health remains controversial, and the mechanisms underlying this relationship are unclear. We aimed to examine the association between motor function and long-term cognitive trajectories and further explore the underlying mechanisms using brain MRI.

**Methods:**

In the Rush Memory and Aging Project, a prospective cohort study, a total of 2,192 volunteers were recruited from the communities in northeastern Illinois and followed up for up to 22 years (from 1997 to 2020). Individuals with dementia, disability, missing data on motor function at baseline, and missing follow-up data on cognitive function were excluded. At baseline, global motor function was evaluated using the averaged *z* scores of 10 motor tests covering dexterity, gait, and hand strength; the composite score was tertiled as low, moderate, or high. Global and domain-specific cognitive functions—including episodic memory, semantic memory, working memory, visuospatial ability, and perceptual speed—were measured annually through 19 cognitive tests. A subsample (n = 401) underwent brain MRI scans and regional brain volumes were measured. Data were analyzed using linear mixed-effects models and linear regression.

**Results:**

Among the 1,618 participants (mean age 79.45 ± 7.32 years) included in this study, baseline global motor function score ranged from 0.36 to 1.82 (mean 1.03 ± 0.22). Over the follow-up (median 6.03 years, interquartile range 3.00–10.01 years), low global motor function and its subcomponents were related to significantly faster declines in global cognitive function (β = −0.005, 95% CI −0.006 to −0.005) and each of the 5 cognitive domains. Of the 344 participants with available MRI data, low motor function was also associated with smaller total brain (β = −25.848, 95% CI −44.902 to −6.795), total white matter (β = −18.252, 95% CI −33.277 to −3.226), and cortical white matter (β = −17.503, 95% CI −32.215 to −2.792) volumes, but a larger volume of white matter hyperintensities (β = 0.257, 95% CI 0.118–0.397).

**Discussion:**

Low motor function is associated with an accelerated decline in global and domain-specific cognitive functions. Both neurodegenerative and cerebrovascular pathologies might contribute to this association.

## Introduction

Motor function is an important indicator of an individual's functional ability.^1^ Age-related motor impairments—including diminished dexterity, slower gait speed, poor coordination, and decreased muscle strength—are common and associated with poorer quality of life and a higher risk of all-cause mortality.^2-4^ Given that motor function can be heterogeneous, the integration of multiple motor measures may help to more comprehensively examine their association with cognitive function changes among older adults.^5^

Growing evidence points to an association between motor impairment and cognitive decline among older adults.^6-13^ However, the relationship between different components of motor function and cognition, including specific cognitive domains, needs to be clarified. Previous studies have related impairments in individual motor indicators (including gait speed and hand strength) to a faster decline in memory function, visuospatial ability, and processing speed.^9-11^ However, other studies found no association between the loss of hand strength and memory, attention, or processing speed.^12,13^ In light of these mixed findings, the association between motor function and long-term cognitive trajectories remains unclear. In addition, previous studies on this topic were limited by relatively short follow-up time and the use of basic cognitive assessments or only a single indicator of motor function.

The mechanisms underlying the association between motor function and changes in cognitive function warrant further investigation. MRI has been widely applied to examine structural brain changes and can provide insight into the pathophysiology underlying changes in cognition.^14^ Previous neuroimaging studies reported an association between motor function and reduced gray matter, white matter, and hippocampal volumes,^15-19^ though other studies produced conflicting results.^15,16^ So far, few studies have systematically addressed the association between motor function and domain-specific cognitive trajectories and brain structural measures assessed by MRI scans.

Within the Rush Memory and Aging Project (MAP), we have previously reported that poor motor function is associated with incident cognitive impairment.^5,8,17^ In this study, we aimed to (1) assess the longitudinal associations between motor function (including dexterity, gait, and hand strength) and trajectories of global and domain-specific cognitive functions and (2) explore the association between poor motor function and structural brain differences. We hypothesize that poor motor function is related to accelerated declines in cognitive function and that neurodegeneration and cerebrovascular pathologies might underlie this association. In this study, we tested these hypotheses using 22-year follow-up data from MAP, a prospective cohort study including brain MRI and comprehensive assessments of motor function and cognitive function over time.

## Methods

### Study Population

**Figure 1:**
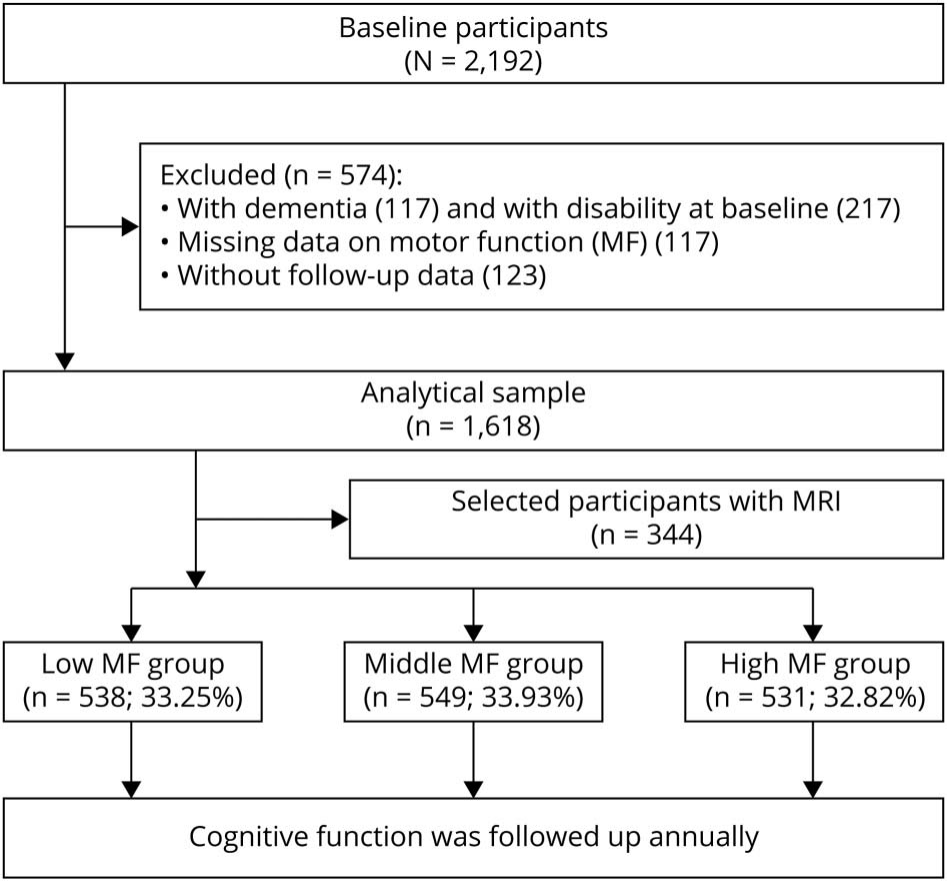
Flowchart of the Study Population MF = motor function.

MAP is an ongoing prospective cohort study focused on cognitive decline and the development of common chronic diseases among older adults. Study design and assessment protocols have been previously described in detail.^18^ In brief, participants were recruited from continuous care retirement communities, senior and subsidized housing, social service agencies, church groups, and individual homes in northeastern Illinois and the Chicago area.^18^ All participants were native English speakers. Between 1997 and 2020, the study included 2,192 participants who were followed up annually for up to 22 years, and 401 of them underwent brain MRI scans between 2009 and 2011. Because MAP is an ongoing open cohort study, MRI scans could occur at baseline for newly recruited participants or during a follow-up examination for already enrolled participants. Thus, the motor function-MRI association is considered cross-sectional. We excluded 574 individuals from the total MAP population, including 117 with no motor function data, 217 with impairment in the activities of daily living (ADL), 117 with prevalent dementia at baseline, and 123 with missing follow-up data on cognitive function. This left a study population of 1,618 participants for this investigation, 344 of whom had MRI data available (Figure 1).

### Standard Protocol Approvals, Registrations, and Patient Consents

The study was approved by the Institutional Review Board of Rush University Medical Center and was conducted in accordance with the ethical standards set forth in the 1964 Declaration of Helsinki and its subsequent amendments. All participants provided written informed consent and repository consent to allow sharing of their data.^19^

### Assessment of Motor Function and ADL

A comprehensive measure of global motor function reflecting dexterity, gait, and hand strength was assessed at baseline using 10 individual performance tests of common motor behaviors.^20^

Motor dexterity was assessed based on 2 tasks: the Purdue pegboard task and the finger-tapping task. In the Purdue pegboard task, participants were required to insert as many nails as possible into the holes of a pegboard within a 30-second period. In the finger-tapping task, participants were required to strike an electric tapper (Western Psychological Services, Los Angeles, CA) with their forefinger as fast as they could for 10 seconds. Each task was repeated 4 times (twice for each hand). The number of nails placed on the pegboard and the number of taps performed in each of the 4 sessions were averaged to obtain an overall Purdue pegboard score and tapping score, respectively, for each participant.

Motor gait was assessed using 2 lower extremity function tasks. Participants were required to walk forward 8 feet and make two 360-degree turns. The time (in seconds) and the number of steps taken for each task were recorded separately and the averages were obtained to yield scores for walking time, walking steps, turning time, and turning steps.

Motor hand strength consisted of 2 dimensions: grip strength and pinch strength. Using the Jamar hydraulic hand and pinch dynamometers (Lafayette Instruments, Lafayette, IN), participants performed a grip and a pinch twice with each hand. Overall grip/pinch strength scores were calculated as the mean grip/pinch strength (in pounds) from these 4 trials.

Balance was assessed by recording the amount of time (up to 10 seconds) participants were able to stand on each leg and their toes. Because the balance tasks were sometimes not attempted, we did not aggregate balance scores into a single measure of overall balance performance.

Given differences in how performance was measured in each of the 10 motor function tests, performance scores for each test were converted to *z* scores and averaged together to obtain a composite measure of global motor function, with higher scores indicating better motor performance.^5^ Global motor function score was tertiled to yield 3 equally sized groups reflecting different levels of motor function (low, moderate, and high).

Disability was assessed using the self-reported Katz ADL scale, which covers 6 basic physical abilities including eating, toileting, bathing, dressing, transferring from bed to chair, and walking across a small room.^21^ Participants who reported difficulty in 1 or more items were considered to have a disability and were excluded from the study population.

### Assessment of Cognitive Function, Mild Cognitive Impairment, and Dementia

Cognitive function was assessed using a battery of 19 cognitive performance tests administered at baseline and annual follow-up visits.^22^ The 19 tests captured 5 cognitive domains, including episodic memory (Word List Memory, Word List Recognition, Word List Recall, Immediate and Delayed Recall of Story A from Logical Memory and East Boston Story), semantic memory (Verbal Fluency, Boston Naming Test, National Adult Reading Test), working memory (Digit Ordering, Digit Span Forward and Backward), perceptual speed (Symbol Digit Modalities Test, Number Comparison, Stroop Word Reading, and Stroop Color Naming Test), and visuospatial ability (Judgment of Line Orientation, Standard Progressive Matrices). Raw scores from all tests were converted to *z* scores and averaged to generate a composite measure of global cognitive function, with higher scores reflecting better cognitive function.^23^

Dementia and mild cognitive impairment (MCI) were diagnosed based on a standard procedure that included computerized scoring of the 19 aforementioned cognitive tests, clinical evaluation by a neuropsychologist, and diagnostic classification by a clinician.^24,25^ Dementia was diagnosed according to the National Institute of Neurological and Communicative Disorders and Stroke and the Alzheimer's Disease and Related Disorders Association (NINCDS/ADRDA) joint working group criteria.^24^ Participants were diagnosed with MCI if they showed evidence of cognitive impairment in the neuropsychologist's examination (i.e., impairment in at least 1 cognitive domain based on normative cognitive data considering age, sex, and education), but did not meet the NINCDS/ADRDA criteria for dementia in the clinician's examination.^25^

### Assessment of Structural Brain Volumes

The MAP MRI protocol is described in eMethods 1 (links.lww.com/WNL/D98). In this study, we obtained the volumes (in cubic centimeters) of the whole brain, total gray matter (cerebellar gray matter, cortical gray matter, and subcortical gray matter), total white matter (cerebellar white matter, cortical white matter), the hippocampus, and white matter hyperintensities (WMH).

### Assessment of Other Variables

During enrollment, participants underwent a comprehensive clinical assessment and physical assessment performed by trained staff and provided information on demographic characteristics, socioeconomic status, daily lifestyle, and medical history. Information on the assessment of education level, body mass index (BMI), alcohol consumption, smoking, physical activity, social activity, cardiometabolic diseases (CMDs; i.e., diabetes, heart disease, or stroke), hypertension, depression, and *APOE* ε4 carrier status are described in detail in eMethods 2 (links.lww.com/WNL/D98). More detailed information about the data collection can be found at the Rush Alzheimer's Disease Center Resource Sharing Hub.

### Statistical Analysis

Baseline characteristics of the study population were compared according to tertiles of motor function. One-way analysis of variance or Kruskal-Wallis rank sum tests were used for continuous variables, and Chi-square tests were used for categorical variables.

Linear mixed-effects models were used to estimate β coefficients and 95% CIs for the association between motor function (both as a continuous and a categorical variable) and annual changes in global cognitive function and 5 cognitive domains. The fixed effect included motor function, quadratic follow-up time (year^2^), and their interaction based on the distribution of the observed association. The random effect included random intercepts and slopes, allowing for the individual differences at baseline and during follow-up. We additionally assessed the joint effect of motor function and other factors of interest on changes in global cognition over follow-up time by creating dummy variables based on the joint exposure of global motor function (low vs high) and CMDs (absence vs presence), *APOE* genotype (ε4 vs no ε4), or physical activity (low vs high). Next, we examined statistical interactions by creating indicator variables containing global motor function, CMDs/*APOE* genotype/physical activity, and their cross-product term in the same model. Finally, linear regression models were used to estimate the β coefficients and 95% CIs for the association between global motor function and structural brain volumes.

All models were adjusted for age, sex, education, BMI, alcohol consumption, smoking, physical activity, social activity, hypertension, diabetes, heart diseases, stroke, depression, and *APOE* ε4 carrier status.

In the sensitivity analysis, we repeated the original analyses after (1) excluding individuals with MCI at baseline, (2) excluding measures of cognitive function assessed during the first 5 years of follow-up to minimize reverse causality, (3) imputing missing values for the covariates using multiple imputation by chained equation, (4) additionally adjusting for global cognition at baseline, and (5) stratifying by sex. A 2-tailed *p* value <0.05 was considered to be statistically significant for all tests. All statistical analyses were performed using Stata SE 15.0 for Windows (StataCorp, College Station, TX).

### Data Availability

Requests for access to MAP data can be made at radc.rush.edu.

## Results

### Characteristics of the Study Population

Of the 1,618 participants included in the study (mean age 79.45 ± 7.32 years, 74.17% female), the global motor function score ranged from 0.36 to 1.82 (mean 1.03 ± 0.22) at baseline. There were 538 (33.25%), 549 (33.93%), and 531 (32.82%) participants in the low, moderate, and high motor function groups, respectively.

Participants with moderate/low motor function were more likely to be older, be female, have less formal education, and have higher BMI compared with those with high global motor function. In addition, participants with moderate/low motor function tended to drink less alcohol, tended to have less physical and social activities, tended to have poorer cognitive performance, and were more likely to have hypertension, heart disease, and stroke. No significant differences among the motor function groups were found in terms of *APOE* ε4 carrier status, smoking, diabetes, or depression (Table 1).

**Table 1:**
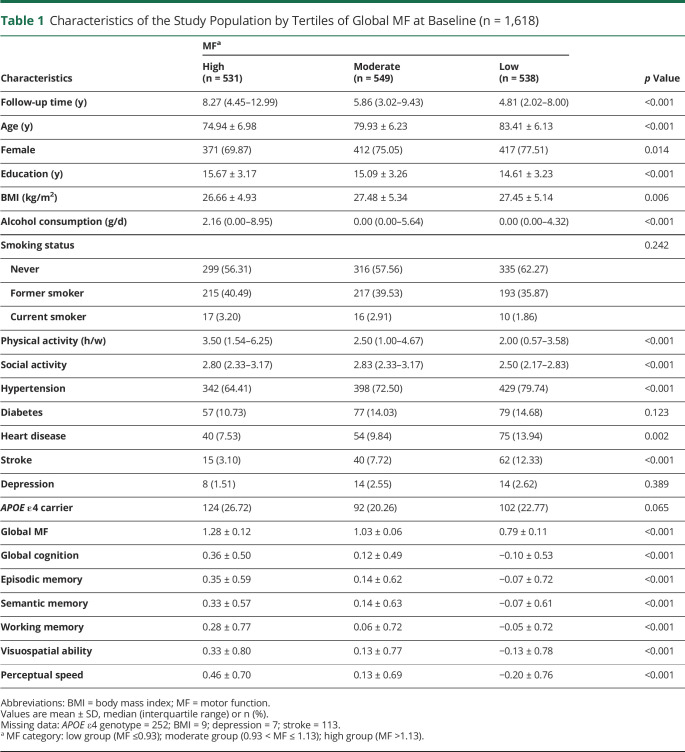
Characteristics of the Study Population by Tertiles of Global MF at Baseline (n = 1,618)

Characteristics	MF^a^			*p* Value
	High (n = 531)	Moderate (n = 549)	Low (n = 538)	
Follow-up time (y)	8.27 (4.45–12.99)	5.86 (3.02–9.43)	4.81 (2.02–8.00)	<0.001
Age (y)	74.94 ± 6.98	79.93 ± 6.23	83.41 ± 6.13	<0.001
Female	371 (69.87)	412 (75.05)	417 (77.51)	0.014
Education (y)	15.67 ± 3.17	15.09 ± 3.26	14.61 ± 3.23	<0.001
BMI (kg/m^2^)	26.66 ± 4.93	27.48 ± 5.34	27.45 ± 5.14	0.006
Alcohol consumption (g/d)	2.16 (0.00–8.95)	0.00 (0.00–5.64)	0.00 (0.00–4.32)	<0.001
Smoking status				0.242
Never	299 (56.31)	316 (57.56)	335 (62.27)	
Former smoker	215 (40.49)	217 (39.53)	193 (35.87)	
Current smoker	17 (3.20)	16 (2.91)	10 (1.86)	
Physical activity (h/w)	3.50 (1.54–6.25)	2.50 (1.00–4.67)	2.00 (0.57–3.58)	<0.001
Social activity	2.80 (2.33–3.17)	2.83 (2.33–3.17)	2.50 (2.17–2.83)	<0.001
Hypertension	342 (64.41)	398 (72.50)	429 (79.74)	<0.001
Diabetes	57 (10.73)	77 (14.03)	79 (14.68)	0.123
Heart disease	40 (7.53)	54 (9.84)	75 (13.94)	0.002
Stroke	15 (3.10)	40 (7.72)	62 (12.33)	<0.001
Depression	8 (1.51)	14 (2.55)	14 (2.62)	0.389
*APOE* ε4 carrier	124 (26.72)	92 (20.26)	102 (22.77)	0.065
Global MF	1.28 ± 0.12	1.03 ± 0.06	0.79 ± 0.11	<0.001
Global cognition	0.36 ± 0.50	0.12 ± 0.49	−0.10 ± 0.53	<0.001
Episodic memory	0.35 ± 0.59	0.14 ± 0.62	−0.07 ± 0.72	<0.001
Semantic memory	0.33 ± 0.57	0.14 ± 0.63	−0.07 ± 0.61	<0.001
Working memory	0.28 ± 0.77	0.06 ± 0.72	−0.05 ± 0.72	<0.001
Visuospatial ability	0.33 ± 0.80	0.13 ± 0.77	−0.13 ± 0.78	<0.001
Perceptual speed	0.46 ± 0.70	0.13 ± 0.69	−0.20 ± 0.76	<0.001

Abbreviations: BMI = body mass index; MF = motor function.

Values are mean ± SD, median (interquartile range) or n (%).

Missing data: *APOE* ε4 genotype = 252; BMI = 9; depression = 7; stroke = 113.

a MF category: low group (MF ≤0.93); moderate group (0.93 < MF ≤ 1.13); high group (MF >1.13).

Moreover, we further compared the baseline characteristics of participants with (n = 344) and without (n = 1,274) MRI, and the analysis showed no significant differences between the 2 groups in terms of sex, education, BMI, alcohol consumption, smoking, hypertension, diabetes, heart disease, stroke, depression, or *APOE* ε4 carrier status (all *p* > 0.05) (eTable 1, links.lww.com/WNL/D98).

### Association of Motor Function With Cognitive Decline

**Figure 2:**
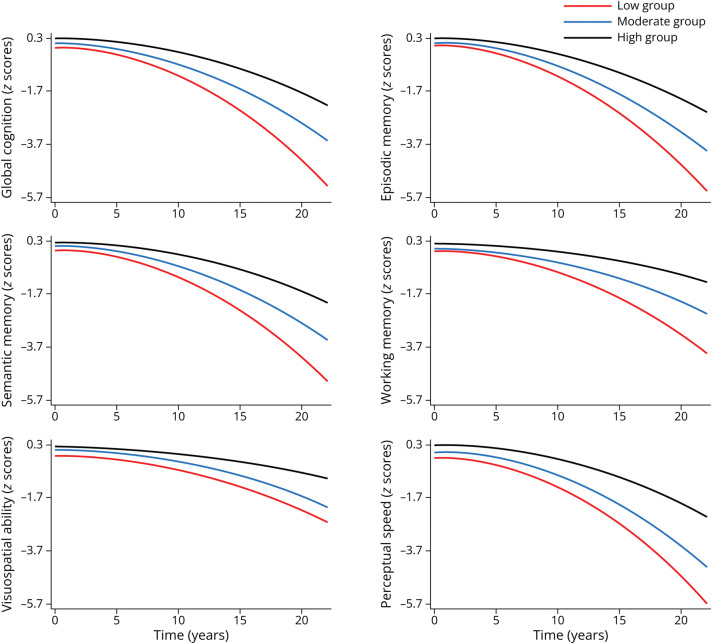
Cognitive Trajectories in Global Cognition and Specific Domains by MF in Tertiles Trajectories represent β coefficients from linear mixed-effect models adjusted for age, sex, education, body mass index, apolipoprotein E epsilon 4, alcohol consumption, smoking, physical activity, social activity, hypertension, diabetes, heart disease, stroke, and depression with the highest MF group as reference group. MF = motor function.

During the follow-up (median 6.03 years, interquartile range 3.00–10.01 years), better global motor function and its subcomponents including dexterity, gait, and hand strength (as continuous variables) were associated with slower declines in global cognition, episodic memory, semantic memory, working memory, visuospatial ability, and perceptual speed over time. When motor function and its subcomponents were analyzed as categorical variables, participants with moderate/low motor function showed significantly faster declines in global cognitive function and each of the 5 domain-specific cognitive functions over time, compared with those with high motor function (Table 2 and Figure 2).

**Table 2:**
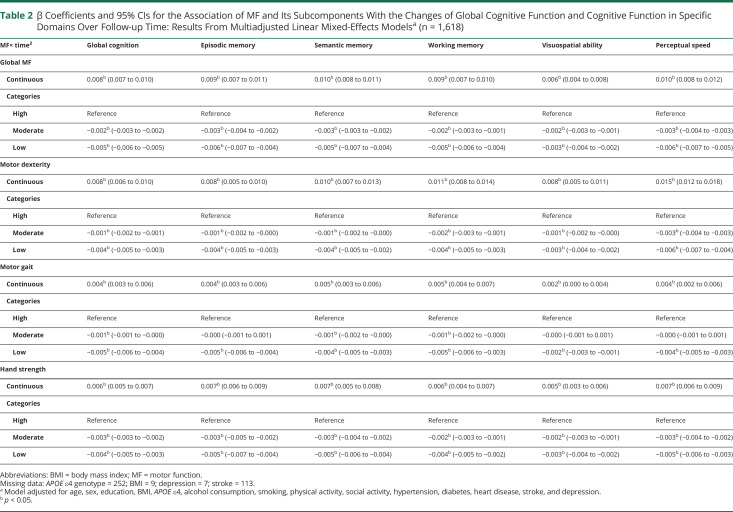
β Coefficients and 95% CIs for the Association of MF and Its Subcomponents With the Changes of Global Cognitive Function and Cognitive Function in Specific Domains Over Follow-up Time: Results From Multiadjusted Linear Mixed-Effects Models^a^ (n = 1,618)

MF× time^2^	Global cognition	Episodic memory	Semantic memory	Working memory	Visuospatial ability	Perceptual speed
Global MF						
Continuous	0.008^b^ (0.007 to 0.010)	0.009^b^ (0.007 to 0.011)	0.010^b^ (0.008 to 0.011)	0.009^b^ (0.007 to 0.010)	0.006^b^ (0.004 to 0.008)	0.010^b^ (0.008 to 0.012)
Categories						
High	Reference	Reference	Reference	Reference	Reference	Reference
Moderate	−0.002^b^ (−0.003 to −0.002)	−0.003^b^ (−0.004 to −0.002)	−0.003^b^ (−0.003 to −0.002)	−0.002^b^ (−0.003 to −0.001)	−0.002^b^ (−0.003 to −0.001)	−0.003^b^ (−0.004 to −0.003)
Low	−0.005^b^ (−0.006 to −0.005)	−0.006^b^ (−0.007 to −0.004)	−0.005^b^ (−0.007 to −0.004)	−0.005^b^ (−0.006 to −0.004)	−0.003^b^ (−0.004 to −0.002)	−0.006^b^ (−0.007 to −0.005)
Motor dexterity						
Continuous	0.008^b^ (0.006 to 0.010)	0.008^b^ (0.005 to 0.010)	0.010^b^ (0.007 to 0.013)	0.011^b^ (0.008 to 0.014)	0.008^b^ (0.005 to 0.011)	0.015^b^ (0.012 to 0.018)
Categories						
High	Reference	Reference	Reference	Reference	Reference	Reference
Moderate	−0.001^b^ (−0.002 to −0.001)	−0.001^b^ (−0.002 to −0.000)	−0.001^b^ (−0.002 to −0.000)	−0.002^b^ (−0.003 to −0.001)	−0.001^b^ (−0.002 to −0.000)	−0.003^b^ (−0.004 to −0.003)
Low	−0.004^b^ (−0.005 to −0.003)	−0.004^b^ (−0.005 to −0.003)	−0.004^b^ (−0.005 to −0.002)	−0.004^b^ (−0.005 to −0.003)	−0.003^b^ (−0.004 to −0.002)	−0.006^b^ (−0.007 to −0.004)
Motor gait						
Continuous	0.004^b^ (0.003 to 0.006)	0.004^b^ (0.003 to 0.006)	0.005^b^ (0.003 to 0.006)	0.005^b^ (0.004 to 0.007)	0.002^b^ (0.000 to 0.004)	0.004^b^ (0.002 to 0.006)
Categories						
High	Reference	Reference	Reference	Reference	Reference	Reference
Moderate	−0.001^b^ (−0.001 to −0.000)	−0.000 (−0.001 to 0.001)	−0.001^b^ (−0.002 to −0.000)	−0.001^b^ (−0.002 to −0.000)	−0.000 (−0.001 to 0.001)	−0.000 (−0.001 to 0.001)
Low	−0.005^b^ (−0.006 to −0.004)	−0.005^b^ (−0.006 to −0.004)	−0.004^b^ (−0.005 to −0.003)	−0.005^b^ (−0.006 to −0.003)	−0.002^b^ (−0.003 to −0.001)	−0.004^b^ (−0.005 to −0.003)
Hand strength						
Continuous	0.006^b^ (0.005 to 0.007)	0.007^b^ (0.006 to 0.009)	0.007^b^ (0.005 to 0.008)	0.006^b^ (0.004 to 0.007)	0.005^b^ (0.003 to 0.006)	0.007^b^ (0.006 to 0.009)
Categories						
High	Reference	Reference	Reference	Reference	Reference	Reference
Moderate	−0.003^b^ (−0.003 to −0.002)	−0.003^b^ (−0.005 to −0.002)	−0.003^b^ (−0.004 to −0.002)	−0.002^b^ (−0.003 to −0.001)	−0.002^b^ (−0.003 to −0.001)	−0.003^b^ (−0.004 to −0.002)
Low	−0.004^b^ (−0.005 to −0.003)	−0.005^b^ (−0.007 to −0.004)	−0.005^b^ (−0.006 to −0.004)	−0.004^b^ (−0.005 to −0.002)	−0.003^b^ (−0.004 to −0.002)	−0.005^b^ (−0.006 to −0.003)

Abbreviations: BMI = body mass index; MF = motor function.

Missing data: *APOE* ε4 genotype = 252; BMI = 9; depression = 7; stroke = 113.

a Model adjusted for age, sex, education, BMI, *APOE* ε4, alcohol consumption, smoking, physical activity, social activity, hypertension, diabetes, heart disease, stroke, and depression.

b *p* < 0.05.

In the joint effect analyses (eTable 2, links.lww.com/WNL/D98), we combined the moderate and low global motor function groups because both were significantly related to cognitive decline. The association between poor global motor function and changes in global cognition over time was stronger among people with CMDs (β = −0.004, 95% CI −0.005 to −0.003). However, after excluding participants with CMDs, the relationship between poor motor function and faster decline in cognitive function still remained significant in people without CMDs (eTable 3). There was a remarkable multiplicative interaction between global motor function and CMDs on cognitive decline (*p* < 0.001). We failed to find the interactions between global motor function and *APOE* ε4 carrier status or physical activity (all *p* > 0.05) (eTables 4 and 5).

### Association of Motor Function With Structural Brain Differences

In the low, moderate, and high motor function groups, 74, 107, and 163 participants, respectively, underwent an MRI scan. The median and interquartile range of the intervals between the baseline examination and the MRI scan was 2.96 (0.00–5.58), 3.87 (0.00–6.02), 3.79 (0.00–6.04) years in each group, and the difference in interval time was not statistically significant (*p* = 0.468). In multiadjusted linear regression models, greater global motor function (as a continuous variable) was related to larger total brain volume (β = 50.995, 95% CI 12.561–89.430) and smaller WMH volume (β = −0.476, 95% CI −0.758 to −0.194). When global motor function was treated as a categorical variable, compared with high motor function, low motor function was related to smaller total brain volume (β = −25.848, 95% CI −44.902 to −6.795), total white matter volume (β = −18.252, 95% CI −33.277 to −3.226), and cortical white matter volume (β = −17.503, 95% CI −32.215 to −2.792), but a larger volume of WMH (β = 0.257, 95% CI 0.118–0.397) (Table 3). The associations between global motor function and gray matter or hippocampal volume were not significant.

**Table 3:**
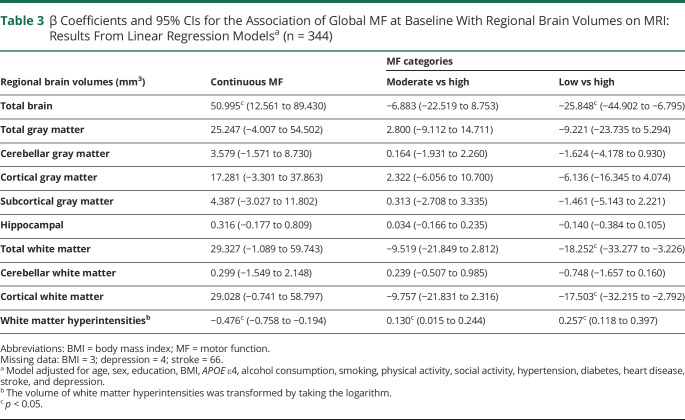
β Coefficients and 95% CIs for the Association of Global MF at Baseline With Regional Brain Volumes on MRI: Results From Linear Regression Models^a^ (n = 344)

Regional brain volumes (mm^3^)	Continuous MF	MF categories	
		Moderate vs high	Low vs high
Total brain	50.995^c^ (12.561 to 89.430)	−6.883 (−22.519 to 8.753)	−25.848^c^ (−44.902 to −6.795)
Total gray matter	25.247 (−4.007 to 54.502)	2.800 (−9.112 to 14.711)	−9.221 (−23.735 to 5.294)
Cerebellar gray matter	3.579 (−1.571 to 8.730)	0.164 (−1.931 to 2.260)	−1.624 (−4.178 to 0.930)
Cortical gray matter	17.281 (−3.301 to 37.863)	2.322 (−6.056 to 10.700)	−6.136 (−16.345 to 4.074)
Subcortical gray matter	4.387 (−3.027 to 11.802)	0.313 (−2.708 to 3.335)	−1.461 (−5.143 to 2.221)
Hippocampal	0.316 (−0.177 to 0.809)	0.034 (−0.166 to 0.235)	−0.140 (−0.384 to 0.105)
Total white matter	29.327 (−1.089 to 59.743)	−9.519 (−21.849 to 2.812)	−18.252^c^ (−33.277 to −3.226)
Cerebellar white matter	0.299 (−1.549 to 2.148)	0.239 (−0.507 to 0.985)	−0.748 (−1.657 to 0.160)
Cortical white matter	29.028 (−0.741 to 58.797)	−9.757 (−21.831 to 2.316)	−17.503^c^ (−32.215 to −2.792)
White matter hyperintensities^b^	−0.476^c^ (−0.758 to −0.194)	0.130^c^ (0.015 to 0.244)	0.257^c^ (0.118 to 0.397)

Abbreviations: BMI = body mass index; MF = motor function.

Missing data: BMI = 3; depression = 4; stroke = 66.

a Model adjusted for age, sex, education, BMI, *APOE* ε4, alcohol consumption, smoking, physical activity, social activity, hypertension, diabetes, heart disease, stroke, and depression.

b The volume of white matter hyperintensities was transformed by taking the logarithm.

c *p* < 0.05.

### Sensitivity Analysis

We obtained similar results after (1) excluding 402 participants with baseline MCI (eTables 6 and 7, links.lww.com/WNL/D98), (2) excluding measurements of cognitive function collected during the first 5 years of follow-up (n = 878) (eTable 8), (3) imputing missing data for covariates (n = 374) (eTable 9), and (4) further adjusting for global cognition at baseline (eTables 10 and 11). After stratifying by sex, the association between motor function and cognitive decline was still significant in both male and female participants (eTables 12 and 13), but the association between poor global motor function and smaller white matter volume and larger WMH volume was significant only in female participants, possibly owing to the smaller sample size (n = 87) of male participants (eTables 14 and 15).

## Discussion

In this community-based cohort study of older adults, we found that (1) low motor function (including dexterity, gait, and hand strength) was associated with accelerated declines in global cognition, episodic memory, semantic memory, working memory, visuospatial ability, and perceptual speed and (2) low global motor function was further related to smaller total brain, total white matter, and cortical white matter volume, but larger WMH volume.

Longitudinal studies have shown that the deterioration of motor dexterity, gait, and hand strength is related to global cognitive decline among older adults.^6-8^ However, previous studies on the association between motor function and domain-specific cognitive function have shown mixed results.^9-13^ Two previous studies have reported significant relationships between slower gait speed and reduced memory performance and visuospatial ability.^9,10^ A systematic review indicated that weakened hand strength was related not only to impaired memory but also a poorer performance on tests of language and processing speed.^11^ However, another study reported no association between motor function and cognitive decline in any domain.^12^ Moreover, one previous investigation even reported that stronger hand strength was related to less decline in memory, spatial ability, and processing speed in people older than 65 years, though this association was not significant among individuals younger than 65 years.^13^ In this study, we combined dexterity, gait, and hand strength as a comprehensive assessment of global motor function and evaluated global cognitive function in addition to 5 cognitive domains. We found that low motor function was related to more rapid decreases in global cognitive function and all 5 domain-specific cognitive functions.

Some studies have linked poor motor function to severe brain atrophy or lesions in people with specific neurologic disorders, such as Alzheimer disease, Parkinson disease, and multiple sclerosis.^26-28^ However, the evidence on the relationship between specific motor functions and structural brain difference has been limited.^15,16,29-31^ Poorer motor dexterity has been associated with larger white matter lesions.^29^ Moreover, gait disorders have been related to smaller white matter, gray matter, and hippocampal volumes.^30,31^ A British birth cohort suggested that reduced hand strength was associated with smaller total brain volume,^15^ while another study on people with dementia showed that hand strength was associated only with smaller left hippocampal volume.^16^ In this study, we demonstrated that poor global motor function was related to smaller total brain, total white matter, and cortical white matter volumes and larger WMH volume. Reduction in total brain volume is a typical marker of neurodegeneration,^32^ whereas reduction in white matter volume can reflect vascular damage to the brain.^33^ In addition, WMH are considered to be a typical MRI expression of microvascular lesions in cerebral white matter.^33^ Our findings suggest that both neurodegeneration and vascular lesions in the brain may underlie the motor function-cognitive decline association.

It is well known that impairment in certain cognitive domains can reflect specific changes in the brain structure. Poor performance in semantic memory, working memory, visuospatial ability, and processing speed is associated with reduced total brain atrophy and an increase in the volume of white matter lesions, such as WMH.^34-37^ Decline in working memory and processing speed might be related to white matter atrophy.^38,39^ Furthermore, poorer performance in perceptual speed and global cognition have been associated with smaller total brain volume.^40^ Thus, the 2 sections of this study—on cognitive domains and brain structural changes—complement each other and strengthen the evidence of a connection between motor function and cognition.

Higher prevalence of diabetes or cardiovascular diseases among people with poor motor function could be one possible explanation for the association between poor motor function and cognitive decline. Type 2 diabetes, heart disease, and stroke have been related to cognitive impairment and irreversible brain pathologies and are prevalent in people with poor motor function.^41-43^ Indeed, we found a significant joint effect of poor global motor function and CMDs on cognitive decline, whereby the poor motor function-cognitive decline association was stronger among people with CMDs. Our finding suggests that CMDs could be a potential factor of risk for faster cognitive decline among older adults, particularly those with diminished motor function. Motor function is an important predictor of vascular risk, which could cause cognitive decline through increased pulsatile cerebral blood flow velocity and decreased cerebral perfusion.^44,45^ Moreover, poor motor function could lead to reduced glucose metabolism and the development of insulin resistance. Insulin resistance in the CNS could trigger cognitive impairment by promoting the phosphorylation of tau proteins.^46,47^ Further long-term studies are warranted to confirm the interaction between poor motor function and CMDs on cognitive decline, given the small sample size in our joint exposure analysis. Notably, in our study, the relationship between poor motor function and faster decline of cognitive functions was still significant among CMD-free participants, so the motor function-cognition association cannot be completely explained by the higher prevalence of CMDs among people with poor motor function. Indeed, poor motor function could cause chronic low-grade local or systemic inflammation by affecting the secretion of cytokines and other peptides, thus resulting in cognitive decline.^48-50^

As a longitudinal community-based cohort study, our study has the advantage of a long follow-up period and a relatively large sample size. In addition, we used a composite measure of motor function and assessed global and domain-specific cognitive functions through a comprehensive battery of 19 tests. Although many studies have reported an association between motor function and cognitive function, no study so far has incorporated brain MRI data, which could provide evidence regarding the mechanisms underlying the motor-cognition association. In addition to the poor motor function-cognitive decline association, we found that poor motor function was associated with brain MRI markers indicative of neurodegeneration and vascular lesions, suggesting the involvement of both neurodegenerative and vascular pathways in the motor-cognition association. Nevertheless, some limitations of our study should be pointed out. First, the study population was older (mean age 79.45 years), was approximately 70% female, and composed of predominantly highly educated people living in urban communities who thus performed better than the general population on cognitive tests. Thus, caution is required when generalizing our findings to other (especially younger) populations. Second, because the exclusion of people with missing data on motor function and cognition may have resulted in a comparatively healthier study population, the observed association between motor function and cognitive function might have been underestimated. However, we repeated the analysis after multiple imputations, and the results were consistent with those in from the initial analysis. Third, composite motor function score was based on *z* scores of 10 individual motor tests and thus may have limited clinical application. Future studies are warranted to develop a standard cutoff in motor function that can be more clinically relevant. Fourth, the relationship between motor function and regional brain volumes could not be examined longitudinally; thus, the temporality of this association is unclear. Moreover, we considered only MRI-derived structural brain volumes, and future imaging studies using modalities such as functional MRI and diffusion tensor imaging are warranted to further explore the mechanisms underlying the association between motor function and cognition. Fifth, the diagnoses of some clinical disorders were based on participants' retrospective self-reports; thus, information bias could not be ruled out. Finally, although we considered a wide range of potential confounders, detailed information on other factors of interest (e.g., hearing, duration and amount of smoking, and the diagnosis of movement disorders such as ataxia and dystonia) was unavailable in the study.

In summary, our study provides evidence that poor motor function is associated with accelerated decline in global cognition, episodic memory, semantic memory, working memory, visuospatial ability, and perceptual speed over time. Neurodegeneration and vascular lesions in the brain might underlie the motor function-cognitive decline association. Future large population-based cohort studies are warranted to corroborate our findings.

## Appendix Authors

**Table:**

Name	Location	Contribution
Zhangyu Wang, MSc	Tianjin Medical University, China	Analyzed and interpreted the data; drafted the article; performed statistical analysis; revised the article for intellectual content
Jiao Wang, PhD	Tianjin Medical University, China	Analyzed and interpreted the data; performed statistical analysis; revised the article for intellectual content
Jie Guo, PhD	Karolinska Institutet, Stockholm, Sweden	Analyzed and interpreted the data; performed statistical analysis; revised the article for intellectual content
Abigail Dove, MSc	Karolinska Institutet, Stockholm, Sweden	Interpreted the data; revised the article for intellectual content
Konstantinos Arfanakis, PhD	Rush University Medical Center, Chicago, IL	Interpreted the data; revised the article for intellectual content
Xiuying Qi, PhD	Tianjin Medical University, China	Revised the article for intellectual content
David A. Bennett, MD	Rush University Medical Center, Chicago, IL	Design and conceptualized study; revised the article for intellectual content; major role in the acquisition of data
Weili Xu, PhD	Tianjin Medical University, China; Karolinska Institutet, Stockholm, Sweden	Design and conceptualized study; interpreted the data; revised the article for intellectual content; major role in the acquisition of data

## Glossary

ADL: activities of daily living
ADRDA: Alzheimer's Disease and Related Disorders Association
BMI: body mass index
CMD: cardiometabolic disease
MAP: Rush Memory and Aging Project
MCI: mild cognitive impairment
NINCDS: National Institute of Neurological and Communicative Disorders and Stroke
WMH: white matter hyperintensities
